# Transmediastinal and Transcardiac Gunshot Wound with Hemodynamic Stability

**DOI:** 10.1155/2014/985097

**Published:** 2014-08-17

**Authors:** Leire Zarain Obrador, Yusef Mohamed Al-Lal, Jorge de Tomás Palacios, Iñaki Amunategui Prats, Fernando Turégano Fuentes

**Affiliations:** Servicio de Cirugía General II, Hospital General Universitario Gregorio Marañón, 28009 Madrid, Spain

## Abstract

Cardiac injuries caused by knives and firearms are slightly increasing in our environment. We report the case of a 43-year-old male patient with a transmediastinal gunshot wound (TGSW) and a through-and-through cardiac wound who was hemodynamically stable upon his admission. He had an entrance wound below the left clavicle, with no exit wound, and decreased breath sounds in the right hemithorax. Chest X-ray showed the bullet in the right hemithorax and large right hemothorax. The ultrasound revealed pericardial effusion, and a chest tube produced 1500 cc. of blood, but he remained hemodynamically stable. Considering these findings, a median sternotomy was carried out, the through-and-through cardiac wounds were suture-repaired, lung laceration was sutured, and a pacemaker was placed in the right ventricle. The patient had uneventful recovery and was discharged home on the twelfth postoperative day. The management and prognosis of these patients are determined by the hemodynamic situation upon arrival to the Emergency Department (ED), as well as a prompt surgical repair if needed. Patients with a TGSW have been divided into three groups according to the SBP: group I, with SBP >100 mmHg; group II, with SBP 60–100 mmHg; and group III, with SBP <60 mmHg. The diagnostic workup and management should be tailored accordingly, and several series have confirmed high chances of success with conservative management when these patients are hemodynamically stable.

Since Ludwig Rehn performed the first successful cardiac injury repair, being able to suture a penetrating wound in the right ventricle, the management of this pathology has drastically evolved.

The main causes of cardiac traumatisms in our environment are motor vehicle collisions. Such traumatisms are a very common finding in autopsy studies of those deceased at the scene [1]. In recent years, due to the improvement of security mechanisms in cars, the frequency of cardiac injuries has decreased. However, there has been a rise of cardiac injuries caused by knives and firearms [2].

We report the case of a 43-year-old male patient with a transmediastinal gunshot wound (GSW) causing cardiopulmonary injuries. When the Emergency Medical Services (EMS) arrived on the scene the patient was conscious, tachypneic, diaphoretic, and hemodynamically stable. He had a GSW in his left infraclavicular region. Bilateral breath sounds were normal, and a decision was made to proceed with orotracheal intubation (OTI). He was taken to our medical center and remained stable during transportation.

Upon arrival to our ED, primary and secondary surveys were carried out according to ATLS protocols, showing decreased breath sounds in the right hemithorax, as well as an entrance wound below the left clavicle, with no exit wound. Chest X-ray showed a bullet in the right hemithorax and a large right hemothorax (Figure 1). A chest tube was inserted, draining 1500 cc. of blood. An echocardiogram revealed a pericardial effusion, with normal cardiac motion. Despite his hemodynamic stability, he was taken straight to the operating room (OR).

A median sternotomy disclosed small hemopericardium, with an entrance wound in the right ventricle (Figure 1) and an exit wound in the right atrium. The bullet had then entered the right chest. Both wounds were suture-repaired with 3.0 Prolene over Teflon pledgets. Small bleeding lung laceration was sutured, and a pacemaker was placed in the right ventricle. The patient needed inotropics during his first hours of ICU admission, but they were discontinued after 24 hours. He remained with good cardiac contractility, and cardiac septal defects were subsequently ruled out. He developed right pneumonia which was successfully managed with antibiotics and was discharged home on the twelfth postoperative day.

Cardiac GSW is associated with high mortality, and its prognosis depends on fast surgical repair. In the past decades, most patients could not reach the hospital alive. However, in recent years, the morbidity and mortality related to this pathology have dramatically decreased due to the advances in prehospital care and the reduction of the transfer time of the patient [3]. The EMS decision for prehospital OTI in our patient seems to have been ill-advised in view of his vital signs and for fear of tension pneumothorax developing after OTI and manual ventilation.

The management and prognosis of patients with cardiac GSW are determined by the hemodynamic situation upon arrival to the ED (Table 1). According to some authors, more than half of the patients who come to the ED after suffering a transmediastinal wound are hemodynamically stable [3, 4]. Up to 60–70% of the stable patients will not need surgery and will be treated in a conservative way [5] once the relevant diagnostic procedures are carried out. CT angiography is considered the gold standard diagnostic procedure in stable patients [4, 6].

The most frequently affected chamber in penetrating cardiac trauma is the right ventricle, which is involved in half of the cases.

The preferred surgical approach for this type of injuries is a median sternotomy, given the excellent exposure and access to all mediastinal structures. In extremely urgent situations, a left anterolateral thoracotomy is recommended, as it allows very fast and direct access to the heart [2].

According to different publications, patients with a transmediastinal GSW can be divided into three groups [3, 5, 7, 8], each of them with different diagnostic and therapeutic management (Table 1).

According to Burack et al. [4], among the 207 patients with mediastinal penetrating wound that were assisted at the ED, 35% were hemodynamically unstable. 26% of these patients died in the ED, while 53 patients were operated urgently, surviving 32. 65% of the patients were stable and underwent a CT angiography, which was normal in 80% of cases, and those patients were managed conservatively.

According to a prospective study by Demetriades and Velmahos [7], conservative management was performed in up to 60% of hemodynamically stable patients with a transmediastinal wound, once the relevant diagnostic procedures were carried out. Both series confirm the high chances of conservative management in patients with hemodynamic stability in this situation.

Nevertheless, a high proportion of patients will ultimately need surgery despite initial hemodynamic stability. This will usually be prompted by the results of imaging techniques and/or thoracic drain output, such as in our case.

## Figures and Tables

**Figure 1:**
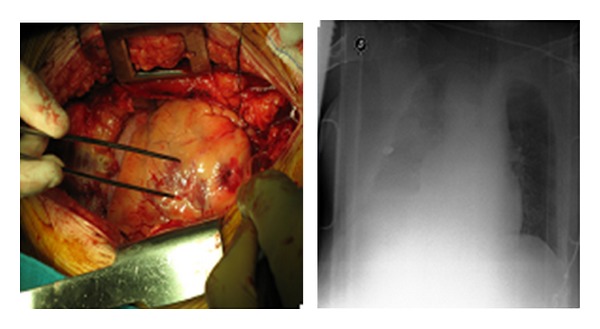
GSW to the right ventricle. Chest X-ray showing a bullet in the right hemithorax and a massive right hemothorax.

**Table 1:** Management of transmediastinal gunshot wounds.

Groups	SBP (mmHg)	Evaluation	Management
I	>100	Chest X-ray, echocardiogram, and CT angiography	(a) Observation: most frequent (b) Surgery
II	60–100	Physical examination +/− chest X-ray and/or echocardiogram if possible	(a) Observation (b) Surgery: most frequent
III	<60	ED thoracotomy	
